# Gastrointestinal Microbiota and Parasite-Fauna of Wild *Dissostichus eleginoides* Smitt, 1898 Captured at the South-Central Coast of Chile

**DOI:** 10.3390/microorganisms9122522

**Published:** 2021-12-07

**Authors:** Italo Fernández, Patricio de Los Ríos-Escalante, Ariel Valenzuela, Paulina Aguayo, Carlos T. Smith, Apolinaria García-Cancino, Kimberly Sánchez-Alonso, Ciro Oyarzún, Víctor L. Campos

**Affiliations:** 1 Departamento de Microbiología, Facultad de Ciencias Biológicas, Universidad de Concepción, Casilla 160-C, Concepción 4070386, Chile; itfernan@udec.cl (I.F.); csmith@udec.cl (C.T.S.); apgarcia@udec.cl (A.G.-C.); kimsanchez@udec.cl (K.S.-A.); 2 Departamento de Ciencias Biológicas y Químicas, Facultad de Recursos Naturales, Universidad Católica de Temuco, Temuco 4780000, Chile; prios@uct.cl; 3 Núcleo de Estudios Ambientales, Universidad Católica de Temuco, Temuco 4780000, Chile; 4 Laboratorio de Piscicultura y Patología Acuática, Facultad de Ciencias Naturales y Oceanográficas, Universidad de Concepción, Concepción 4070386, Chile; avalenz@udec.cl (A.V.); coyarzun@udec.cl (C.O.); 5 Institute of Natural Resources, Faculty of Veterinary Medicine and Agronomy, Universidad de Las Américas, Sede Concepción, Chacabuco 539, Concepción 3349001, Chile; paulinaaguayo@udec.cl; 6 EULA Environmental Sciences Center, Faculty of Environmental Sciences, Universidad de Concepción, Concepción 4070386, Chile

**Keywords:** *Dissostichus eleginoides*, *Nototheniidae*, microbiota, parasite-fauna

## Abstract

*Dissotichus eleginoides* has a discontinuous circumpolar geographic distribution restricted to mountains and platforms, mainly in Subantarctic and Antarctic waters of the southern hemisphere, including the Southeast Pacific, Atlantic and Indian oceans and in areas surrounding the peninsular platforms of subantarctic islands. The aim of this work was to determine and characterize the gastrointestinal parasitic and microbial fauna of specimens of *D. eleginoides* captured in waters of the south-central zone of Chile. The magnitude of parasitism in *D. eleginoides* captured in waters of the south-central zone of Chile is variable, and the parasite richness is different from that reported in specimens from subantarctic environments. Next-generation sequencing (NGS) of the microbial community associated to intestine showed a high diversity, where *Proteobacteria, Firmicutes*, and *Bacteriodetes* were the dominant phyla. However, both parasitic and microbial structures can vary between fish from different geographic regions

## 1. Introduction

The Family *Nototheniidae* comprises numerous species of fish that mainly inhabit Antarctic and subantarctic waters [1]. Within this family, the Patagonian toothfish *Dissostichus eleginoides* Smitt, 1898, also known as Chilean Sea Bass, stands out because it is considered one of the main target species of commercial fishing in the Southern Ocean.

*D. eleginoides* has a discontinuous circumpolar geographic distribution restricted to mountains and platforms, mainly in subantarctic and Antarctic waters of the southern hemisphere, including the Southeast Pacific, Atlantic and Indian oceans and in areas surrounding the peninsular platforms of subantarctic islands. In its projection towards the South American cone, it is distributed along the continental slope to Peru (6° LS) through the Pacific and Uruguay (35° LS) through the Atlantic, bordering the entire area of Patagonia at depths ranging between 80 and 2500 m [2].

*D. eleginoides* is a benthopelagic high trophic level (secondary and/or tertiary) carnivorous predator fish. It is capable to ascend in the water column with a minimal energy expense to feed itself and it shows seasonal and geographic variations in its diet [3]. Its growth is slow and shows a late maturity and low fecundity [2,4]); characteristics which make it particularly susceptible to overexploitation. In the 90s, the volumes captured worldwide reached year averages exceeding 40,000 tons but captures have gradually decreased to approximately 20,000 tons per year [2,5]. In Chile, the current legislation regulating its exploitation limits the areas allowed for its capture and establishes a close season between June and August, coinciding with the spawning period for this species [6]. In the year 2019, the total capture in Chile, including that set aside for research purposes, was only 4271 tons [7]. Presently, the length (head-tail) size of fish captured ranges from 60 to 120 cm, although it is possible to find larger specimens (exceeding 2 m and 100 kg) in sub-Antarctic waters. This fish is considered a high-quality product, and it is exported mainly to the Asiatic market. It is exported eviscerated (fresh or frozen) or in more processed forms, such as eviscerated without the head, fish fillets, and also smoked [8].

Various contributions to the knowledge of its biology have been reported in recent years, although insufficiently known relevant aspects still persist [9,10,11,12,13,14]. Microbiological background knowledge of this species is very scarce, and it is limited to what has been recently reported regarding the bacterial microbiota of the digestive tract of this species [15]. From samples isolated from the gastrointestinal tract of a specimen of *D. eleginoides* which was captured in southern waters and kept confined for six months, microbiological analysis was carried out by means of traditional culture techniques and identification by 16SrRNA sequencing. However, these results do not reflect the total microbiological diversity, therefore, a study using molecular methods using tools such as next-generation sequencing (NGS), which allows us to obtain a broader microbiological profile, is necessary.

On the other hand, although the parasitic fauna of *D. eleginoides* has been more studied, the background on specimens captured in Chilean waters is also scarce and they go back more than a decade ago. This data accounts for the presence of 11 parasitic taxa, exclusively gastrointestinal, in specimens captured in the south-central zone of Chile [16,17,18,19,20,21,22].

Given these antecedents, the aim of this work was to determine and characterize the gastrointestinal parasitic and microbiological fauna of specimens of *D. eleginoides* captured in waters of the south-central zone of Chile.

## 2. Materials and Methods

### 2.1. Sampling

Specimens of wild *D. eleginoides* were collected during commercial fishing campaigns carried out between 2019 and 2020 in the south-central zone of Chile (37°–39° S; average depth: 920 m). Fishing and fish management is regulated by the general law of fishing and aquaculture of Chile, Decree N° 8892. Taxonomic affiliation of the fish was performed according to Oyarzún (2003) [23]. From 47 specimens of *D. eleginoides*, skin (swabbing for microbiology and direct inspection of the entire surface, including the mouth, fins, and gills, to detect the presence of ectoparasites) and digestive tube samples were obtained. additionally, random samples of hypaxial-epaxial muscle, for parasitological analysis, were obtained (specimens for commercial use, after sampling were returned to the production chain, CEBB 1020-2021). To avoid loss of gastrointestinal content, the oesophageal and anal ends were tied. Later, samples were transported at 4 °C to the Parasitology Laboratory of the Faculty of Biological Sciences (Universidad de Concepción, Concepcion, Chile) where the posterior area of the stomach was additionally tied, to separate the stomach from the intestine. Samples for microbiology, skin, stomach, and intestine were obtained in triplicate and frozen at −40 °C using RNAlater (Thermo Fisher Scientific, Waltham, MA, USA) to stabilize and protect the samples for subsequent molecular analysis. Seawater samples were obtained using an oceanographic rosette equipped with 10 L Niskin bottles (General Oceanic, Miami, FL, USA).

### 2.2. Isolation and Identification of Parasites

To detect protozoa, a sample of the stomach and intestinal contents was collected from each portion of the digestive tract and processed using the modified Burrows sedimentation technique [24]. The rest of the content of each sample was extracted and sieved, separately, with saline solution under pressure in a plastic cylinder whose bottom contained a 0.50 mm mesh. The material retained by the sieves was examined using a stereomicroscope (Zeiss Stemi DRC, 4×, Jena, Germany), which allowed the isolation of parasite specimens. In addition, endoparasites were detected and extracted from the mucosa, submucosa, and serosa of the gastrointestinal tract and from the adjacent mesenteries. Moreover, the musculature of the *D. eleginoides* specimens was checked to detect parasites that could have zoonotic importance. All parasites were fixed in 70% alcohol before their taxonomic analysis. The specimens of Nematodes and Digenea were diaphanized with Amman’s lactophenol to visualize their internal structures. Prior to staining, Cestodes and Trematodes were decolorized, dehydrated, and diaphanized, and then stained with Harris’s hematoxylin and mounted in Canada balsam. Taxonomic identification was carried out using optical microscopy (Motic, BA 310, 10× and 40×, Kowloon Bay, Kowloon, Hong Kong) and specialized reference literature [25,26,27]. The typified specimens were deposited at the Parasitology Museum of Universidad de Concepción (MPUDEC, Reg. DE1-9).

### 2.3. Characterization of the Parasite Community

The descriptors of magnitude, prevalence, and the mean abundance of the parasitism were calculated according to Bush et al. [28]. For each infra-community, the total abundance (total of parasitic individuals of all taxa) and richness (number of parasites taxa) were calculated according to Holmes & Price [29]. The diversity of parasites was calculated using Shannon Weaver (H′), Margalef, and Pielou indexes. Dominance was evaluated using the Simpson index [30]. The non-parametric index, Chao1, was used to calculate the parasitic abundance.

### 2.4. Characterization of the Microbial Community

#### 2.4.1. PCR-DGGE and Analysis of DGGE Profiles of the Microbial Community

Total DNA from skin, stomach, and intestine was extracted using the E.Z.N.A. DNA/RNA Isolation Kit (Omega BioTek, Norcross, GA, USA), following the protocol provided by the manufacturer. 16S rRNA universal primers EUB 9-27 and EUB 1542 were used for amplification of DNA [31]. Then, Nested PCR was performed using the primer pair 341f and 534r with a GC clamp (CGCCC GCCGC GCGCG GCGGG CGGGG CGGGG GCACG GG GGG) according to Cuevas et al. [32]. DGGE was performed with a DGGE 1001 system (C.B.S. Scientific Company Inc., San Diego, CA, USA) according to Campos et al. [33].

For DGGE profiles analyses, DGGE gels were digitized using a photo-documentation system MaestroGen (MaestroGen Inc., Hsinchu, Xiangshan, Taiwan). For the analysis of banding profiles, a binary matrix was constructed based on the presence (1) or absence (0) of individual bands in each lane using the Gel-Pro Analyzer 4.0 software package (Media Cybernetics, Silver Spring, MD, USA). A multidimensional scaling diagram (MDS) was constructed using the Bray Curtis algorithm, according to Cuevas et al. [32].

#### 2.4.2. Genomic DNA Extraction and Massive Sequencing of the Microbial Community

Genomic DNA was extracted from the skin, stomach, and intestine, of three *D. eleginoides* specimens, using the E.Z.N.A. DNA/RNA Isolation Kit (Omega BioTek, Norcross, GA, USA), following the protocol provided by the manufacturer. The DNAs extracted were subsequently purified using the UltraClean 15 DNA Purification Kit (MoBio, Carlsbad, CA, USA). Quality and concentration of DNAs were checked by UV/Vis spectroscopy (NanoDrop ND-1000, Peq- lab, Erlangen, Germany). For DNA extraction of water samples, 2 L of seawater were pre-filtered through a 20 μm pore size mesh, then the biomass was collected onto 0.22 μm pore size PES filters as described for the natural communities by Aguayo et al. [34].

Total DNA extracted from the skin, stomach, and intestine (pool of three *D. eleginoides*) and seawater were quantified and sequenced. Illumina Miseq sequencing was performed at Genoma Mayor, Universidad Mayor, Santiago, Chile. 16S rARN raw data was analyzed using the Mothur software (version 1.35.1, Ann Arbor, MI, USA). Short reads (less than 200 bp) were discarded and sequences that were likely due to errors and assemble reads which differed by only 2bp were removed using the “pre.cluster” (read denoised) command in Mothur’s platform. UCHIME algorithm was used to identify and remove chimeric sequences and the remaining sequences were classified using the SILVA database [35].

### 2.5. Data Analyses and Diversity Indices

Data were analyzed using two-way ANOVA and Student’s *t*-test using the GraphPad Prism 5 software. *p* values < 0.05 were considered as statistically significant [31]. Diversity indexes and PCA analyses were carried out using PRIMER 6.1.18 (Primer-E, Ltd., Auckland, New Zealand) and non-parametric analyses were carried out using the R software version 3.1.0 (R-GNU project, Auckland, New Zealand). [36]. In addition, the prevalence and mean intensity of infection, and the quantitative descriptors of the magnitude and the parasite richness, as a community descriptor, were ecologically characterized according to Bush et al. [28].

## 3. Results

### 3.1. Parasite-Fauna Community Composition Analysis

#### 3.1.1. Parasitic Structure

All specimens examined were parasitized by at least one parasitic taxon (Table 1). The parasitic Isopod *Rocinela* aff. *australis* (Schiœdte & Meinert, 1879), adult stages of Digenea, *Brachyphallus crenatus* (Rudolphi, 1802; Odhner, 1905), *Derogenes varicus* (Müller, 1784; Loos, 1901), *Neolepidapedon* spp. (Manter, 1954), *Gonocerca* spp. (Manter, 1925), *Lecitochirium* spp. (Lühe, 1901) and the nematode *Hysterothylacium* spp. (Ward and Magath, 1917), were identified and classified into genera or species levels. Furthermore, larval stages of the Cestode *Hepatoxylon trichiuri* (Holten, 1802), and of the Nematodes *Anisakis* spp. (Dujardin, 1845) and *Pseudoterranova* spp. (Mozgovoi, 1953) were detected. The presence of Protozoa was not detected in the gastrointestinal tracts analyzed.

#### 3.1.2. Parasite-Fauna Diversity Estimates

The results showed that *Anisakis* spp. (100%) and *Lecithochirium* spp. (82.9%) exhibited the highest prevalence. Only *R.* aff *australis*, *Pseudoterranova* spp., *Derogenes varicus* and *Gonocerca* spp., presented prevalences lower than 50% (Table 1). A total of 3414 parasitic specimens were detected and distributed in 10 taxa. Of these, 1850 corresponded to Plathelminthes (Digenea: 1765; Cestodes: 85) and 156 to Nematodes. Only one arthropod specimen was detected (Table 1). Also, *Anisakis* spp. and *Pseudoterranova* spp. were detected in 28 (P: 59.6%; MA: 4.4) and 14 (P: 29.8%; MA: 1.0) *D. eleginoides* specimens, respectively.

The ecological indices are shown in Table 2. The specific diversity (Shannon–Wiener and Margalef indexes) was high in the stomach samples. Equity and dominance by Pielou and Simpson indexes were higher in the intestine samples.

### 3.2. Total Microbial Community Composition Analysis

#### 3.2.1. Analysis of Similarity of Bacterial Communities by DGGE

**Figure 1:**
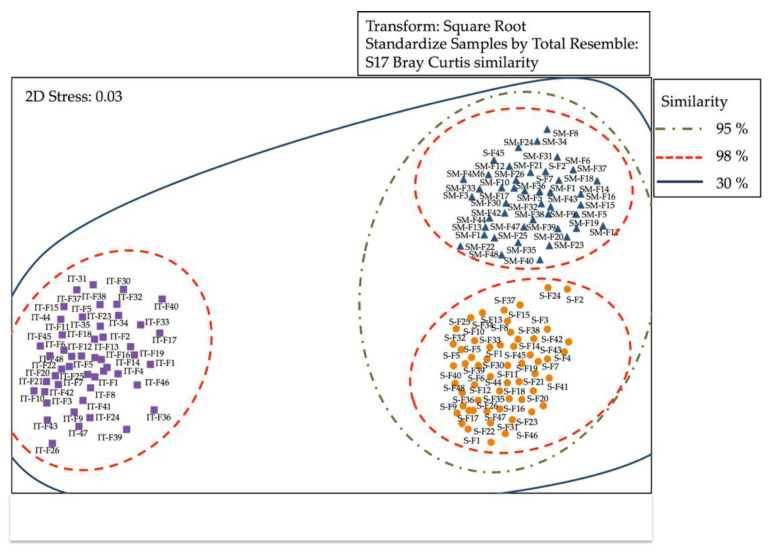
Multidimensional scaling (MDS) of the denaturing gradient gel electrophoresis (DGGE) data matrix of bacterial 16s rRNA from *D. eleginoides*. Stomach (S); skin (SM); intestine (IT).

DGGE profile bands or OTUs were analyzed using the Bray–Curtis correlation (Figure 1). Multidimensional scaling (MDS) analysis of the banding pattern, obtained by DGGE, revealed that there was a high degree of similarity between the replicas of the samples (98% similarity). The similarity percentage for stomach was 30% when compared to the samples of the intestine, where ANOSIM analysis (R = 0.40, *p* = 0.0010) showed that differences between the abundance of OTUs were significant. Similar percentages were detected for skin when compared to the samples of the intestine and the ANOSIM analysis (R = 0.39, *p* = 0.0020) showed significant differences. A high degree of similarity was observed between the stomach and skin, showing non-significant differences in the abundance of OTUs (R = 0.007, *p* = 0.4620). These results were consistent with the hierarchical cluster analysis (Bray–Curtis index), which clearly indicated the higher similarity between replicates for stomach, intestine, and skin samples.

#### 3.2.2. Sequencing Data and Diversity Estimates

The Illumina-based analysis of the universal V1–V2 region of the 16S rRNA genes for Bacteria and Archaea, after quality check within the SILVA database and removing chimeras, a total of 85,210 (99.4%) high-quality sequences remained. Skin samples showed the highest number of quality reads (36.520), and the highest number of OTUs was retrieved from intestinal samples (21,644) (Table 3). The intestine showed the highest Shannon diversity index (H′ = 4.515). Non-parametric Chao1 and ACE estimators predicted that the highest richness was in the intestine, whereas the lowest was in the stomach.

#### 3.2.3. Microbial Diversity Analyses

**Figure 2:**
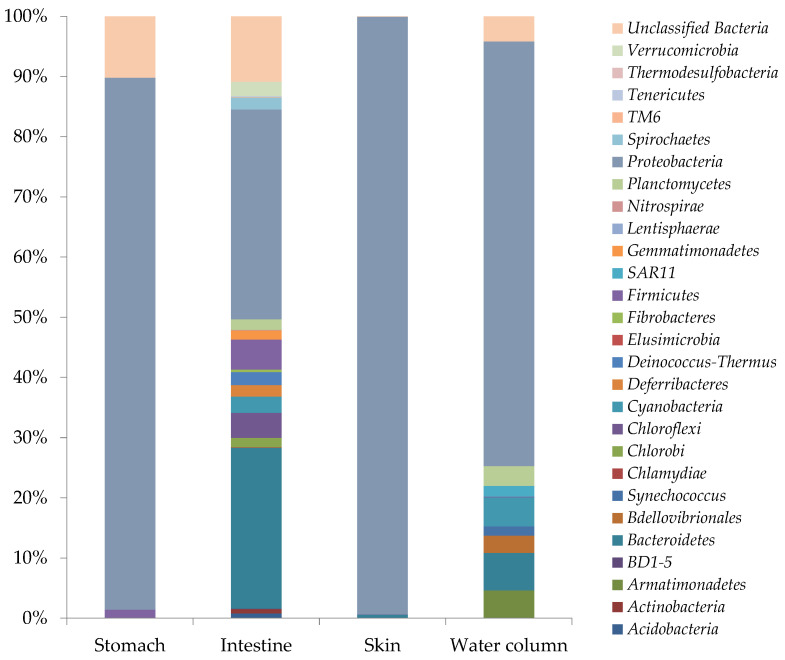
Relative abundance of sequences (percentage) assigned to *Bacteria* and *Archaea* domains phylogenetic groups from skin, stomach, and intestine of *D. eleginoides*.

Retrieved bacterial OTUs were classified in a total of 28 different bacterial phyla, of which *Proteobacteria*, *Firmicutes*, and *Bacteriodetes* presented the highest relative abundances of total bacterial OTUs (Figure 2). Intestine was the sample that presented the highest diversity index (H′ = 3.834), where *Proteobacteria* (34.8%), *Bacteriodetes* (27%), *Chlorobi* (1.5%), *Firmicutes* (5%) *Cloroflexi* (4.1 %), *Cyanobacteria* (2.7%), *Deferribacteres* (1.9%), *Deinococcus-Thermus* (2.1%), *Gemmatimonadetes* (1.6%), *Planctomicetes* (2%), *Spirochaeta* (1.9%) were dominant phyla (≥1%). The skin harbored a higher percentage of *Proteobacteria* (99%) and a low percentage of *Bacteroidetes* and *Firmicutes* (<1%). *Bacteroidetes* phylum was not detected in stomach samples. In the water samples (environmental sample) *Alteromonadales* (50%), *Bdellovibrionales* (10%), *Rhodobacterales* (32%) and clade SAR11 (8.9%) (Figure 2).

*Gamma-proteobacteria* represented the most abundant *Proteobacteria* class in stomach and skin samples (88.2% and 98.9%, respectively). In the intestine, *Alpha-proteobacteria* and *Delta-proteobacteria* were the most abundant subclasses (14.3% and 11.6%), while *Gamma-proteobacteria* (4.91%) and *Beta-proteobacteria* (2.3%) presented the lower percentages of total bacterial OTUs.

Similarly, a highest percentage of *Sphingobacteriia* (10.9%), *Bacteroidia* (6.1%) and *Clostridia* (4.3%) were detected in the intestine. Other OTUs of dominant taxonomic groups (abundances ≥ 1%), were affiliated to *Cytophaga* (3.8%), *Flavobacteria* (1.6%), *Ignavibacteria* (1.4%), *Chloroflexi* (3.2%), *Cyanobacteria* (2.6%), *Deferribacteres* (1.9%), *Deinococci* (2.1%), *Gemmatimonadetes* (1.6%), *Planctomycetacia* (1.3%), *Sphirochaetas* (2.0%), *Opitutae* (1.2%) (Figure 2).

From all samples, 492 genera were retrieved. The highest number of genera was observed in the intestine (466), when compared to stomach (22) and skin (60). A total number of 21 dominant genera (≥1%) were retrieved from the intestine, four from the stomach, and three from the skin. A minor part of dominant bacterial genera (6/97) was ubiquitous in all samples: *Maribacter (Flavobacteriaceae), Synechococcus (Synechococcaceae), Rhodobacter (Rhodobacteraceae), Acidovorax (Comamonadaceae), Pelagibacter*, and *Pseudoalteromonas (Pseudoalteromonadaceae)*. However, different abundant genera were unique for each sample.

On the other hand, three different *Archaea* phyla, *Crenarchaeota*, *Euryarchaeota*, and *Korarchaeota* were retrieved from all samples. However, *Euryarchaeota* was the dominant phylum in all samples. In particular, *Methanobacteria* (21.9%), *Methanococci* (9.1%), *Methanomicrobia* (8.2%), *Thermococci* (1.5%), and *Thermoplasmata* (3.7%) were dominant phyla (≥1%) in the intestine. In the case of the stomach and skin, the relative abundances of *Archaea* were very low (≤1% total *Archaea* OTUs).

## 4. Discussion

From the parasitic and microbiological point of view, ecological investigations of Antarctic and subantarctic fish are scarce. Since its life cycle includes several phases, the ecology of *D. eleginoides* is characterized by being complex. Eastman [37], points out that semi-pelagic juveniles (12–15 cm total length) become demersal reaching 150 to 400 m depth and, after several years, grow to 60–70 cm total length. Later, the adult fish migrate to meso and bathypelagic habitats at depths greater than 1000 m. This situation would determine that throughout its life cycle, the Patagonian toothfish would be exposed to being infected with various forms of life, perhaps explaining its high parasitic diversity [17].

Therefore, it is expected that the conformation of the microbiota and parasite-fauna of *D. eleginoides*, be affected by factors such as the heterogeneity of environments and the occurrence of extensive vertical and horizontal migrations. However, our results showed differences, at the level of parasite richness, prevalence, and abundance, when compared to that reported in other geographical areas studied [17,18,19]. This difference may be the consequence, of its extensive distribution, and that that *D. eleginoides* is not a panmictic population. There are reports that in the South Atlantic there would be different population structures of the species, whose variations could be influenced by migratory movements between feeding and spawning areas [38,39,40]. Diverse interactions established within the trophic web and the types of environments, would explain the conformation of its parasite-fauna. On the contrary, *D. eleginoides* from the coast of the Pacific Ocean would be influenced by the cold Humboldt current, which would generate a stable habitat, with a homogeneous parasitic distribution.

Oliva et al. [22] supported the above assumptions using parasites as biological markers, as a population discriminating tool, suggesting that in the south-central coast of Chile, there is only one stock of *D. eleginoides*. Our results support this hypothesis since the parasite richness and the prevalence and abundance values are like those previously reported in specimens of *D. eleginoides* captured in Chilean waters, except for the larvae of *Pseudoterranova* spp., not previously reported by Rodríguez and George-Nascimento [21] or Oliva et al. [22].

In particular, the conformation of the parasitic fauna and its magnitude in the specimens examined in this study may reflect the top predator role of *D. eleginoides*. In addition, it would determine the structure of the intestinal microbiota, being related to this ecological context, since/because all the parasites reported in the present study, except for *Rocinela* aff. *australis*, were transmitted to the host by consumption of infected prey. Therefore, the quantitative and qualitative variations of these parasitic taxa depend on the rate of encounters between the predator and parasitized prey, that is, on the composition of their diet [41,42].

Murillo et al. [3] indicated that the main prey-items of *D. eleginoides*, captured in three areas of the south-central coast of Chile, were mainly bony fish (Macrouridae and Ophidiidae) and to a lesser extent Cephalopods, and only occasionally Anthozoa and Polychaeta. In this way, although the knowledge of the life cycles of the parasites identified in this study is not sufficiently clarified, the results reported here confirm the participation of *D. eleginoides* as a definitive, intermediate, or paratenic host.

Noble [43] and Campbell et al. [41] reported that polychaetes and crustaceans (especially Copepods and Isopods), would act as intermediate hosts for the Digenea reported in this study, where *D. eleginoides* would participate as the definitive host. Cephalopods could transmit the larvae of nematodes, trematodes, and cestodes, while teleost fish could infect *D. eleginoides* with anisakid larvae. In this way, Patagonian toothfish would participate as an intermediate and/or paratenic host of anisakids, infecting the definitive hosts of these parasites, such as marine mammals and sharks, potential and recognized predators of *D. eleginoides* [21,44].

At the infra-population level, the diversity and dominance indices determined that the stomach portion is more diverse when compared to the intestinal portion (preference of this habitat for Digenea) of the digestive tract. The occurrence of anisakid larvae in both digestive portions stands out, representing their recognized flexibility to locate in different habitats within the host.

On the other hand, the absence of Protozoa in the samples is due to the extensive bathymetric migrations, as well as the extreme conditions in which *D. eleginoides* carries out its trophic life. This fact may interrupt the transmission cycle of these microorganisms [17,18,19,20]. This situation also seems to determine a low presence of ectoparasitic taxa in *D. eleginoides*. However, the way of collection and preservation of the samples, during the fishing campaign, could be potential factors that may explain the absence of protozoa and the low prevalence of ectoparasites.

On the contrary, the finding of *Pseudoterranova* spp. larvae, the first in specimens of *D. eleginoides* in Chilean waters, is not by chance since this nematode is a generalist parasite described in numerous hosts that inhabit the Chilean coastline [45]. Furthermore, this genus together with *Anisakis* sp. and *H. trichiuri* have been identified as zoonotic [46], being the ingestion of raw or undercooked fish meat the cause of human infection [47]. This can be supported by the detection of *Anisakis* spp. and *Pseudoterranova* spp. in muscle tissue of *D. eleginoides*. In Chile, the genus *Pseudoterranova* has been linked as the most frequent cause of gastric symptoms in parasitized people [48].

Metagenomic analyses of the microbial community of *D. eleginoides* demonstrated that *Proteobacteria*, *Firmicutes*, and *Bacteroidetes* were the dominant phyla in our study, in agreement with the results reported by other authors [49,50,51]. However, a low bacterial diversity in stomach and skin samples was detected, reduced to the presence of two and one dominant phyla (>1%), respectively. Some authors have proposed that as skin is constantly in contact with the aquatic environment, hence, resulting in a largely synchronized bacteria composition on the skin of the fish and that of the environment. However, a recent study by Aguayo et al. [34] showed a higher diversity in seawater of the Southern Pacific Ocean, and that most of the phyla found are associated with free-living microorganisms, which do not cause infections. In relation to the stomach microbiota, the distribution of microbial communities in this organ may also be largely influenced by the type of diet consumed by the fish.

Alpha diversity of both stomach and skin of *D. eleginoides* was significantly low. The stomach of fish is the first part of the digestive system where the food is subjected to the influence of hydrolytic enzymes and other chemical agents like hydrochloric acid. It leads to lysis of sensitive bacterial cells and subsequent bacterial DNA degradation. This can directly affect the establishment of the bacterial community either due to low pH (HCl) levels and the hydrolytic activity of proteases [49,50]. The low alpha diversity in the skin samples could be explained by the presence of mainly free-living phyla in the seawater samples, which would be unable to colonize the fish and could be mainly involved in the biogeochemical cycles of the ocean.

The analyses at the intestine level of *D. eleginoides* showed a high diversity, agreeing with the one supported by Song et al. [51], who studied, based on 16S rRNA gene sequence through the Illumina MiSeq platform, the composition of the intestinal microbial community of four austral Perciformes species. These authors reported that *Actinobacteria*, *Proteobacteria*, *Firmicutes*, *Thermi*, and *Bacteroidetes* were the most dominant groups at the phylum level. *Firmicutes* and *Bacteroidetes* contribute to carbohydrates and/or proteins fermentation in the intestine to help the host acquire nutrients from the diet. In the case of the *Archaea* domain, *Crenarchaeote* was the most dominant group at the phylum level. Similar results were reported by Wilkins et al. [52], suggesting that environmental factors shape the microbial assembly of the Southern Ocean, and the presence of *Crenarchaeota* in the samples is due to the sub-Antarctic habitat of *D. eleginoides*.

The high relative abundance of *Gamma-proteobacteria*, *Alpha-proteobacteria*, and *Delta-proteobacteria* suggests a beneficial relationship between the gastrointestinal bacterial community and the host because the bacteria identified are involved in nutrient cycling. In addition, carbon cycling and biodegradative capabilities are widespread characteristics within the members of the *Alpha*- and *Gamma-proteobacteria*, including *Pseudomonadaceae*, *Sphingomonadaceae*, *Vibrionaceae*, and the members of the *Cytophaga-Flavobacteria-Bacteroides* (CFB) clade (*Cytophagaceae, Flavobacteriaceae* and *Bacteroidetes*) [53].

Urtubia et al. [16], using culture-dependent techniques, reported that *Vibrio* spp. and *Psychrobacter* spp. were the most frequently recovered bacterial genera at the gastrointestinal level in specimens of *D. eleginoides* captured at the Diego Ramirez Archipelago, (a group of subantarctic islands south of cape Horn). Instead, in our study, *Psychrobacter*, bacteria of cold environments, showed a higher relative abundance in skin samples, evidencing that the cold habitat of *D. eleginoides*, selects a “cold tolerant microbiota”. Since *Pseudomonas*, which also presented a high relative abundance at the skin level, is as well frequently isolated from cold environments, the concept of a “cold tolerant microbiota” is reinforced for *D. eleginoides*.

In general, it is complex to explain the variability of the microbiota at the intestinal level between fish from different geographic regions, since the influence of water and storage conditions [54], DNA extraction protocols [55], diets [56], high level of intraspecific variation [57], sex [58], length/mass of fish [57], time after feeding [59], among other factors, which often influence ecological studies of gastrointestinal bacterial communities.

## 5. Conclusions

This is the first study on parasitology and microbial ecology of *D.* *eleginoides,* a species with economic importance aimed to improve the knowledge about the structure of the microbial and parasitic community of this species. These newly acquired data provide evidence that *D. eleginoides* introduce microbes from the ocean environment and its discontinuous circumpolar geographic distribution can trigger complex interactions between different communities, both microbial and parasitic. Therefore, the functional roles of these taxa associated to different *D. eleginoides* niches have both commercial and ecological interest. It is relevant to emphasize that three parasitic helminths, detected in this study, represent a potential risk of zoonotic transmission through the consumption of raw or insufficiently cooked fish.

## Figures and Tables

**Table 1:** Maturity, prevalence, mean abundance of infection, and parasite collection site of specimens present in *D. eleginoides* captured in waters of the south-central zone of Chile.

Parasite	Adult/Larva	P (%)	MA	Site
ISOPODA				
*Rocinela* aff. *australis*		2.12	0.02	Skin
NEMATODA				
*Anisakis* spp.	L	100	29.17	Stomach/Intestine
*Pseudoterranova* spp.	L	19.14	0.51	Stomach/Intestine
*Hysterothylacium* spp.	A	55.31	3.61	Stomach/Intestine
TREMATODA				
*Brachyphallus crenatus*	A	68.08	10.72	Stomach
*Lecithochirium* spp.	A	82.97	21.23	Stomach
*Derogenes varicus*	A	10.63	0.38	Stomach
*Neolepidapedon* spp.	A	61.7	4.23	Stomach/Intestine
*Gonocerca* spp.	A	8.5	0.97	Stomach
CESTODA				
*Hepatoxylon trichiuri*	L	53.19	1.8	Stomach/Intestine

P: prevalence; MA: mean abundance; L: larvae; A: adult.

**Table 2:** Diversity by means of Shannon (H′), Pielou (j), Simpson (λ), and Margalef indexes of parasites from *D. eleginoides* captured in waters of the south-central zone of Chile.

Indexes	Stomach	Intestine
Shannon (H′)	1.431	1.411
Pielou (j)	0.6514	0.8765
Simpson (λ)	0.7016	0.7445
Margalef	0.9923	0.7287

**Table 3:** Sequencing information, diversity index (H′) and estimator of richness (Chao1 and ACE) obtained after Illumina sequencing.

Indexes	Stomach	Intestine	Skin
Number of high-quality reads	4131	23,703	20,847
Shannon (H′)	0.8837	4.515	0.6971
Dominance_D	0.5921	0.02719	0.6805
Equitability_J	0.2781	0.7211	0.1689
Simpson	0.4079	4.515	0.6971
Margalef	2.86	5.20	0.61
OTUs at 97% (genetic sim)	3085	21,644	20,617
Chao1	263.8	1653.1	149.8

## References

[1] Nelson J., Grande T., Wilson M. (2016). Fishes of the World.

[2] Collins M.A., Brickle P., Brown J., Belchier M. (2010). The Patagonian toothfish: Biology, ecology and fishery. Adv. Mar. Biol..

[3] Murillo C., Oyarzún C., Fernández I. (2008). Variación latitudinal y estacional en la dieta de *Dissostichus eleginoides* Smitt, 1898 (Perciformes: *Nototheniidae*) en ambientes profundos de la costa centro-sur de Chile. Gayana.

[4] Horn P.L. (2002). Age and growth of Patagonian toothfish (*Dissostichus eleginoides*) and Antarctic toothfish (*D. mawsoni*) in waters from the New Zealand subantarctic to the Ross Sea, Antarctica. Fish. Res..

[5] Prenski L.B., Almeyda S.M. (2000). Some biological aspects relevant to patogonian toothfish (*Dissostichus eleginoides*) exploitation in the Agentina exclusive economic zone and adjancent ocean sector. Frente Maritino.

[6] FAO (2004). Patagonian Toothfish (*Dissostichus eleginoides*). www.fao.org/docrep/006/y5261e/y5261e09.html.

[7] Young Z., Gili R., Cid L. (1995). Prospección de Bacalao de Profundidad Entre las Latitudes 43° y 47° S. Informe Técnico.

[8] Servicio Nacional de Pesca y Acuicultura (2020). Control Cuota Pesquería Bacalao de Profundidad (*Dissostichus eleginoides*), año 2019. https://www.subpesca.cl/portal/616/w3-article-826.html.

[9] Subsecretaría de Pesca y Acuicultura (2015). Antecedentes para la Elaboración del Plan de Manejo de las Pesquerias de Bacalao de Profundidad (*Dissostichus eleginoides*). https://www.subpesca.cl/portal/616/articles-103137_documento.pdf.

[10] Arana P. (2009). Reproductive aspects of the Patagonian toothfish (*Dissostichus eleginoides*) off southern Chile. Lat. Am. J. Aquat. Res..

[11] Sellanes J.M., Pedraza-García J., Zapata-Hernández G. (2012). Las áreas de filtración de metano constituyen zonas de agregación del bacalao de profundidad (*Dissostichus eleginoides*) frente a Chile central?. Lat. Am. J. Aquat. Res..

[12] Gallardo P. (2016). Antecedentes preliminares del cultivo de bacalao de profundidad (*Dissostichus eleginoides*; *Nototheniidae*) en la región de Magallanes, Chile. Ann. Inst. Patagon.

[13] Canales C., Ferrada-Fuentes S., Galleguillos R., Oyarzún C., Hernández R. (2018). Population genetic structure of Patagonian toothfish (*Dissostichus eleginoides*) in the Southeast Pacific and Southwest Atlantic Ocean. PeerJ.

[14] Sáez S., Jaramillo R. (2020). Estudio comparativo de escamas de las líneas laterales y corporales del Bacalao de profundidad *Dissostichus eleginoides* (Teleostei: *Nototheniidae*). Rev. Biol. Mar. Ocean.

[15] Troccoli G., Aguilar E., Martínez P., Belleggia M. (2020). The diet of the Patagonian toothfish *Dissostichus eleginoides*, a deep-sea top predator of Southwest Atlantic Ocean. Polar Biol..

[16] Urtubia R., Gallardo P., Cárdenas C., Lavin P., González-Aravena M. (2017). First characterization of gastrointestinal culturable bacteria of Patagonian toothfish *Dissostichus eleginoides* (*Nototheniidae*). Rev. Biol. Mar. Oceanogr..

[17] Gaevskaya A.B., Kovaljova A.A., Parukhin A.M. (1990). Peculiarities and formation of parasitofauna of the Patagonian toothfish *Dissostichus eleginoides*. Biol. Morya.

[18] Brickle P., Mackenzie K., Pike A. (2005). Parasites of the Patagonian toothfish, *Dissostichus eleginoides* Smitt, 1898, in different parts of the sub-Antarctic. Pol. Biol..

[19] Brickle P., Mackenzie K., Pike A. (2006). Variations in the parasite fauna of the Patagonian toothfish (*Dissostichus eleginoides* Smitt, 1898), with length, season, and depth of hábitat around the Falkland Islands. J. Parasitol..

[20] Brown J., Brickle P., Scott B.E. (2013). The parasite fauna of the Patagonian toothfish *Dissostichus eleginoides* off the Falkland Islands. J. Helminthol..

[21] Rodríguez L., George-Nascimento M. (1996). La fauna de parásitos metazoos del bacalao de profundidad *Dissostichus eleginoides* Smitt, 1898 (Pisces: *Nototheniidae*) en Chile central: Aspectos taxonómicos, ecológicos y zoogeográficos. Rev. Chil. Hist. Nat..

[22] Oliva M., Fernández I., Oyarzún C., Murillo C. (2008). Metazoan parasites of the stomach of *Dissostichus eleginoides* Smitt 1898 (Pisces: Notothenidae) from southern Chile: A tool for stock discrimination?. Fish. Res..

[23] Oyarzún C. (2003). Catálogo de los Peces Presentes en el Sistema de Corrientes de Humboldt Frente a Chile Centro Sur.

[24] Muñoz V., Dorn L., Reyes H. (1984). Examen coproparasitológico. Evaluación de algunas modificaciones al método de Burrows (PAF). Parasitol. Día.

[25] Rocka A. (2003). Cestodes of the Antarctic fishes. Polis. Pol. Res..

[26] Rocka A. (2004). Nematodes of the Antarctic fishes. Polis. Pol. Res..

[27] Bray R.A., Gibson D.I., Jones A. (2008). Keys to the Trematoda.

[28] Bush A.O., Lafferty K.D., Lotz J.M., Shostak A.W. (1997). Parasitology meets ecology on its terms: Margolis et al. revisited. J. Parasitol..

[29] Holmes J.C., Price P.W., Anderson D.J., Kikkawa J. (1986). Communities of Parasites. Community Ecology: Patterns and Processes.

[30] Moreno C.E. (2001). Métodos para Medir la Biodiversidad.

[31] Guzmán-Fierro V., Moraga R., León C., Campos V., Smith C., Mondaca M. (2015). Isolation and characterization of an aerobic bacterial consortium able to degrade roxarsone. Int. J. Environ. Sci. Technol..

[32] Cuevas J., Moraga R., Sánchez-Alonzo K., Valenzuela C., Aguayo P., Smith C.T., García A., Fernandez Í., Campos V.L. (2020). Characterization of the Bacterial Biofilm Communities Present in Reverse-Osmosis Water Systems for Haemodialysis. Microorganisms.

[33] Campos V.L., Valenzuela C., Yarza P., Kampfer P., Vidal R., Zaror C., Mondaca M.A., Lopez-Lopez A., Rossello-Mora R. (2010). *Pseudomonas arsenicoxydans* sp. nov., an arsenite-oxidizing strain isolated from the Atacama Desert. Syst. Appl. Microbiol..

[34] Aguayo P., Campos V.L., Henríquez C., Olivares F., De la Iglesia R., Ulloa O., Vargas C.A. (2020). The Influence of *p*CO_2_-Driven Ocean Acidification on Open Ocean Bacterial Communities during A Short-Term Microcosm Experiment in the Eastern Tropical South Pacific (ETSP) off Northern Chile. Microorganisms.

[35] Herrera C., Moraga R., Bustamante B., Vilo C., Aguayo P., Valenzuela C., Smith C., Yanez J., Fierro G.V., Roeckel M. (2021). Characterization of Arsenite-Oxidizing Bacteria Isolated from Arsenic-Rich Sediments, Atacama Desert, Chile. Microorganisms.

[36] R Core Team (2014). Foreign: Read Data Stored by Minitab, S, SAS, SPSS, Stata, Systat, Weka, dBase, R Package Version 0.8–61. http://CRAN.R-project.org/package=foreign.

[37] Eastman J.T. (1993). Antarctic Fish Biology: Evolution in a Unique Environment.

[38] Shaw P.W., Arkhipkin P.W., Al-Khairulla H. (2004). Genetic structuring of Patagonian toothfish populations in the Southwest Atlantic Ocean: The effect of the Antarctic Polar Front and deep-water troughs as barriers to genetic exchange. Mol. Ecol..

[39] Ashford J., Jones C.M., Hofmann E., Everson I., Moreno C., Duhamel G., Williams R. (2005). Can otoliths elemental signatures record the capture site of Patagonian toothfish (*Dissostichus eleginoides*), a fully marine fish in the Southern Ocean. Can. J. Fish. Aquat. Sci..

[40] Laptikhovsky V., Arkhipkin A., Brickle P. (2006). Distribution and reproduction of the Patagonian toothfish *Dissostichus eleginoides* Smitt around the Falkland Islands. J. Fish Biol..

[41] Campbell R., Haedrich R., Munro T. (1980). Parasitism and ecological relationships among deep-sea benthic fishes. Mar. Biol..

[42] Rohde K. (2005). Marine Parasitology.

[43] Noble E. (1973). Parasites and fishes in a deep-sea environment. Adv. Mar. Biol..

[44] Carvajal J. (1974). Records of cestodes from Chilean sharks. J. Parasitol..

[45] Torres P., Hernández E., Sandoval I. (1983). Anisakiasis and phocanemiasis in marine fishes from south of Chile. Int. J. Zoonoses.

[46] Mercado R., Apt W., Castillo D., Akira K., Hiroshi K., Toshiaki K. (2015). *Hepatoxylon trichiuri*. Identificación molecular de un nuevo agente de parasitosis humana en Chile. Parasitol. Lat..

[47] Madrid V., Rivera A., Fernández I. (2016). Prevalencia de larvas de Anisakidae (Nematoda: Ascaridoidae) en musculatura de merluza chilena, *Merluccius* sp. comercializada en Concepción, Chile, en distintos períodos. Parasitol. Lat..

[48] Torres P., Jercic M., Weitz J., Dobrew K., Mercado R. (2007). Human pseudoterranovosis, an emerging infection in Chile. J. Parasitol..

[49] Solovyev M.M., Izvekova G.I., Kashinskaya E.N., Gisbert E. (2018). Dependence of pH values in the digestive tract of freshwater fishes on some abiotic and biotic factors. Hydrobiologia.

[50] Ikeda-Ohtsubo W., Brugman S., Warden C.H., Rebel J.M.J., Folkerts G., Pieterse C.M.J. (2018). How can we define optimal microbiota? A comparative review of structure and functions of microbiota of animals, fish, and plants in agriculture. Front. Nutr..

[51] Song W., Li L., Huang H., Jiang K., Zhang F., Chen X., Zhao M., Ma L. (2016). The Gut Microbial Community of Antarctic Fish Detected by 16S rRNA Gene Sequence Analysis. BioMed Res. Int..

[52] Wilkins D., Van Sebille E., Rintoul S.R., Lauro F.M., Cavicchioli R. (2013). Advection shapes Southern Ocean microbial assemblages independent of distance and environment effects. Nat. Commun..

[53] Nurul A.N.A., Muhammad D.D., Okomoda V.T., Nur A.A.B. (2019). 16S rRNA-Based metagenomic analysis of microbial communities associated with wild *Labroides dimidiatus* from Karah Island, Terengganu, Malaysia. Biotechnol. Rep..

[54] Dehler C.E., Secombes C.J., Martin S.A.M. (2017). Environmental and physiological factors shape the gut microbiota of Atlantic salmon parr (*Salmo salar* L.). Aquacult.

[55] Kashinskaya E.N., Andree K., Simonov E.P., Solovyev M.M. (2017). DNA extraction protocols may influence biodiversity detected in the intestinal microbiome: A case study from wild Prussian carp, *Carassius gibelio*. FEMS Microbiol. Ecol..

[56] Ringø E., Zhou Z., Vecino J.L.G., Wadsworth S., Rinerim J., Krogdahl A., Olsen R.E., Dimitroglou A., Foey A., Davies S. (2016). Effect of dietary components on the gut microbiota of aquatic animals. A never-ending story?. Aquac. Nut..

[57] Vasemägi A., Visse M., Kisand V. (2017). Effect of environmental factors and an emerging parasitic disease on gut microbiome of wild salmonid fish. mSphere.

[58] Bolnick D., Snowberg L., Hirsch P., Lauber C.L., Org Q., Parks B., Lousis A.J., Knight R., Caporaso J.G., Svanback R. (2014). Individual diet has sex-dependent effects on vertebrate gut microbiota. Nat. Commun..

[59] Zhang Z., Li D., Refaey M.M., Xu W. (2017). High spatial and temporal variations of microbial community along the southern catfish gastrointestinal tract: Insights into dynamic food digestion. Front. Microbiol..

